# Psycho-behavioral predictors of uncontrolled blood pressure: A case-control study

**DOI:** 10.34172/hpp.2022.27

**Published:** 2022-08-20

**Authors:** Zeinab Javadivala, Akbar Ranjbarkhah, Asghar Mohammadpoorasl, Farhad Shekari, Devender Bhalla, Neda Gilani

**Affiliations:** ^1^Department of Health Education & Promotion, Tabriz University of Medical Sciences, Tabriz, Iran; ^2^Member of the Student Research Committee, Faculty of Health, Tabriz University of Medical Sciences, Tabriz, Iran; ^3^Department of Statistics and Epidemiology, Faculty of Health, Tabriz University of Medical Sciences, Tabriz, Iran; ^4^Ple Universitaire Euclide Intergovernmental UN Treaty 49006/49007, Bangui, Central African Republic; ^5^Tabriz Health Services Management Research Center, Tabriz University of Medical Sciences, Tabriz, Iran

**Keywords:** Prediction, Blood pressure, Hypertension, Case-Control, Epidemiology

## Abstract

**Background:** We aimed to determine the role of demographic, lifestyle, and personality trait factors in predicting control of blood pressure (BP) among patients with hypertension (HTN) in West Azerbaijan, Iran.

**Methods:** In this case control study we recruited participants from all primary health centers of Salmas city; who were at least 18 years of age, had a HTN diagnosis during the previous six months, and had a mandatory household record. Of 490 random subjects approached, 441 (84.2%) fulfilled our inclusion criteria (case: 221; control: 220). The age-matched controls were recruited from the same source population and were required to have controlled HTN. Data were collected through demographic Checklist, Ten-Item Personality Inventory (TIPI) and International Physical Activity Questionnaire (IPAQ).

**Results:** Upon multivariate analyses, factors related to personality traits subdomains including extraversion personality (odd ratio [OR]: 0.85; CI: 0.73, 0.97) was effective in control of BP. Factors related to uncontrolled BP were agreement and consciences subdomains (OR 1.26 [CI: 1.07, 1.48] and OR 1.21 [CI: 1. 02, 1.44]), rare fruit consumption (OR 5.95 [CI: 1.24, 12.1]), Grade 1 and 2 obesities (OR 2.29 [CI: 1.28, 4.09] and OR 7.11 [CI: 2.21, 12.52]) and smoking (OR 3.27 [CI: 1.56, 6.89]).

**Conclusion:** In addition to regular physical activity and fruit consumption and quitting smoking; personality traits such as Agreement and conscience personality traits were predictive of HTN control. We believe our work provides the required knowledge to design comprehensive HTN prevention programs by taking into account the multi-level causality approach.

## Introduction

 Hypertension (HTN) is a major, independent, and progressive but a preventable disease condition. It is also an important risk factor for other disease conditions, and has been connected with a large assortment of morbidities world over.^1^ For instance, coronary heart disease, heart failure, stroke, myocardial infarction, atrial fibrillation peripheral artery disease, chronic kidney disease, cognitive impairment, and wound healing are associated to the disease. HTN is also the leading single contributor to all-cause mortality and disability worldwide, with about four million deaths every year and one in every eight deaths worldwide.^1^ About half of the world’s adult population is likely to have non-optimal (i.e., >110–115 mm Hg) systolic blood pressure (BP) levels.^2^ The importance of HTN can be further understood from a simple example that a mere ten-point increase in the diastolic pressure above 115/75 mm Hg can double the risk of cardiovascular and cerebrovascular disorders such as stroke.^1^ Therefore, better understanding of its prevention, control, and adequate mitigation is key in promoting health and well-being in world’s population.

 HTN is often projected as a prerogative of the aged population, but, the relationship between BP and age is graded and continuous over one’s life course. For example, age is a proxy of the probability and duration of exposure to numerous factors that increase BP gradually over time, such as excess sodium intake, gain of weight and obesity, alcohol intake, physical inactivity, etc. Others have also shown that atherosclerotic factors are more important than the level of BP alone, since such persons are more likely to derive benefits from interventions.^3^ Beside these factors, there are other important risk factors, for instance, the prevalence of HTN and its consequences are greatest in those with lower socioeconomic status and urban dwellers; a phenomenon seen in both within and between countries.^4^ In the last decade, social, mental, and economic changes in the Eastern Mediterranean and Middle Eastern countries have contributed to a surge of many cardiovascular risk factors, including HTN.^5^

 Unfortunately, despite a grave epidemiological profile for HTN, there remains laxity in patient’s attitudes towards HTN. For instance, in Saudi Arabia, merely 6.2% subjects maintain high adherence to anti-HTN medications^6^ and many people may find hard to accept that HTN may lead to serious consequences.^7^ In Iran, studies show^8^ that the frequency of HTN awareness is merely about 46.0%. The role of patients is more decisive in Middle-Eastern societies, where people tend to retain their strong ethnic identity, and tend to integrate religion and find Islamic values in treatment modalities, which in turn affect their attitude and help-seeking practices.^9^ Another reason behind the patient’s lax attitude towards HTN could be related to one’s personality traits. For instance, personality factors are likely to be associated with incidence and clinical diagnosis of HTN,^10^ as well as with adherence to treatment in patients with chronic conditions.^10,11^ The importance of patient-level factors and patient’s own role in the management of HTN can also be understood from the definition of adherence, which is the extent to which a person’s behaviour- taking medication, following a diet, and/or executing lifestyle changes, corresponds with agreed recommendations from a health care provider.

 Although many studies have explored factors in cross-sectional designs^10^ but, the predictive value of risk and protective factors can only be determined thru’ case-control designs. So, the primary objective was to determine the role of demographic, lifestyle, and personality trait factors in predicting the control of BP among patients with arterial hypertension in West Azerbaijan, Iran.

## Materials and Methods

###  Design and participants

 In this case control study we recruited our participants from all primary health centers of Salmas, West Azerbaijan. Salmas is located northwest of Lake Urmia, near Turkey from March to August, 2019. It has a population of about 127 864. The ethnic identity of the population is mainly Azerbaijanis and Kurds. For this study, the participants were required to have a formal diagnosis of HTN made anytime during the previous six calendar months, irrespective of their current anti-hypertensive treatment status. Uncontrolled HTN was defined as an “average arterial BP measured at the time of clinic visit to be ≥140/90 mm Hg in patients on treatment (at least one anti-hypertensive for minimum two weeks)”.^12,13^ Other inclusion criteria for our study were that the participants be at least 18 years of age and have a household health record in any of the primary health centers of Salmas. Those with diabetes and/or cognitive disorders were excluded from the study. In Iran, the federal government has established health centers throughout the country. These centers are required to maintain a mandatory household file for each household under their respective catchment area. The health centers are run by a general physician and health technicians. The health centers monitor and guide health houses, and provide out-patient care and referral to the district hospitals.

###  Sampling

 The sample size was determined based on the findings related to BMI of two groups of controlled BP and uncontrolled BP of the study by Arabzadeh et al.^14^ By considering these indices; 95% confidence level, and power of 80%, sample size was calculated at 147 per each groups by G-Power 3.1.2 software^15^ (available at: https://www.psychologie.hhu.de/arbeitsgruppen/allgemeine-psychologie-und-arbeitspsychologie/gpower). Considering a design effect of 1.5 and a dropout rate of 10%, the final sample size was determined to be 245 subjects for each group. For this study, we approached a random sample of 490 subjects for inclusion; of which, 441 (84.2%) subjects fulfilled our inclusion criteria and consented as well to participate (case: 221, control: 220). Initially, all subjects were contacted over telephone to check their eligibility and interest to participate, as well as for inviting them to come to the nearest health center for formal clinical assessment and data collection. The age-matched controls (n= 220) were recruited from the same source population and were required to have controlled HTN, i.e. an average arterial BP of <140/90 mm Hg at the clinic visit in patients on treatment (at least one anti-hypertensive for minimum two weeks).

###  Data collection

 Data were collected by administering a demographic Checklist, Ten-Item Personality Inventory (TIPI) and International Physical Activity Questionnaire (IPAQ). Furthermore, patients’ BP, waist circumference (WC), weight, and body mass index (BMI) were also recorded. BP was measured with a mercury sphygmomanometer, twice in the same arm, after the participant had been seated at rest for 10–15 minutes. The systolic and diastolic BP measurements were the mean of the two readings. WC was evaluated using a measuring tape to the nearest 0.1 cm. The weight of an individual dressed in light clothing without shoes was recorded each time using a calibrated scale to the nearest 0.1 kg. Height was measured without shoes using a stadiometer to the nearest 0.1 cm.

###  Ten-Item Personality Inventory 

 TIPI^16^ is a very short measure of the Big Five personality traits (openness, conscientiousness, extraversion, agreeableness, and emotional stability). Each personality dimension is measured by two items. All items are rated on a 7-point Likert-type scale ranging from 1 (Strongly disagree) to 7 (Strongly agree). The total score may range between 10 and 70. Higher scores indicated better personality traits. TIPI has been validated in Iranian population and is found to have adequate validity and reliability.^17^ The content and face validity of the TIPI were assessed qualitatively by panel of 10 experts. Cronbach’s alpha was 0.85, indicating good internal consistency for TIPI.

###  International Physical Activity Questionnaire 

 The Persian version of IPAQ was used to measure regular PA.^18,19^ Information on the time spent on low, moderate and high activities on the basis of METs (Metabolic equivalents)-min/week’s scores or the frequency of activities at week days and time spent on each activity. The calculations of MET’s scores and PA classifications are revealed in the guidelines and other studies.^19,20^

###  Statistical analyses 

 Statistical analyses were conducted using Stata statistical package version 16.^21^ Data were presented using frequencies and proportion for categorical variables and mean and standard deviation for normal numeric variables. Inferential statistics were calculated to compare the characteristics of patients with controlled and uncontrolled HTN. Between-group differences were calculated using Pearson chi-square; and with independent *t* tests. Univariable logistic regression was used to investigate the unadjusted effect of each variable in prediction of uncontrolled BP. Also, a stepwise multivariable logistic regression of reduced number of predictor variables was performed to build the best logistic regression model of uncontrolled BP (critical level of *P* < 0.1 for new variable entry and of *P* ≥ 0.2 for variable removal). The odds ratios (ORs) and 95% confidence intervals (CIs) were also calculated from logistic regression analyses. The model assumptions including collinearity (i.e. wide confidence intervals) and the presence of outliers were checked and were not in violation. The fit of the logistic regression model was confirmed by the Hosmer-Lemeshow goodness-of-fit test. Inferential statistical tests were considered significant when *P* values were less than 0.05.

## Results

 Among 441 participants, 310 were females (70.3%) and 131 (29.3%) were males; 210 (40.30%) were <60 and 231 (52.4%) were ≥60 years of age; 287 (65.1%) were illiterate, and 113 (25.6%) were educated at the primary level. Also, 354 (80.3%) were married and the majority (324, 73.5%) had a low-income level. Among uncontrolled BP, most (57.1%) were ≥60 years of age, and those with uncontrolled BP were older by an average 4.2 years. uncontrolled BP differed in terms of BMI and WC abnormalities, smoking status, use of table salt, type of cooking oil used, consumption of dairy products and fruits. Specific details are provided in Table 1.

**Table 1:** Participants’ demographic characteristic and status of healthy behavior

**Variables**		**Blood pressure control status:** **441 (100), N(%)**		***P*** ** value**
		**Yes** **220 (49.9)**	**No** **221 (50.1)**	
Gender	Female	156 (50.3)	154 (49.7)	0.778^a^
	Male	64 (48.9)	67 (51.1)	
Age (y)	<60	121 (57.6)	89 (42.4)	0.002^a^
	≥60	99 (42.9)	132 (57.1)	
	Mean (SD)	58.08 (12.7)	62.22 (12.7)	0.001^c^
Education level	Illiterate	130 (45.3)	157 (54.7)	0.124^b^
	Primary	66 (58.4)	47 (41.6)	
	Secondary	22 (60.55)	15 (39.45)	
	University	2 (50)	2 (50)	
Marital status	Single	41 (47.1)	46 (52.9)	0.565^a^
	Married	179 (50.6)	175 (49.4)	
Income	< 2 a Month	160 (49.2)	164 (50.8)	0.435^a^
	2-4 a month	55 (53.3)	48 (46.7)	
	>4 a month	5 (38.5)	9 (61.5)	
Job	Employee	4 (66.7)	2 (33.3)	0.962^a^
	Un employee	158 (51.6)	61 (48.4)	
	Housewife	158 (50)	158 (50)	
BMI	Normal	72 (64.3)	40 (35.7)	<0.001^b^
	Overweight	86 (52.1)	79 (47.9)	
	Grade 1 obesity	57 (41.6)	80 (58.4)	
	Grade 2 obesity	5 (18.5)	22 (81.5)	
Waist (cm)	<90	92 (61.3)	58 (38.7)	<0.001^a^
	≥90	128 (44.3)	163 (55.7)	
Daily smoking	No	204 (52.2)	187 (47.8)	0.007^a^
	Yes	16 (32)	34 (68)	
Use table salt	Always	6 (26.1)	17 (73.9)	0.005^a^
	Sometimes	66 (44)	84 (56)	
	Seldom	148 (55.2)	120 (44.8)	
Type of oil consumed	Oil only solid semi-solid or animal	19 (44.2)	24 (55.8)	0.001^a^
	A combination of liquid and solid	130 (45)	159 (55)	
	Liquid plant only	71 (65.1)	38 (34.9)	
Consumption of fast food or carbonated beverages	2 a week	1 (33.3)	2 (66.7)	0.443^a^
	1-2 a month	45 (45)	55 (55)	
	Rarely/Never	174 (51.5)	164 (48.5)	
Daily milk and dairy	Rarely	2 (28.6)	5 (71.4)	0.085^b^
	<2 shares	76 (44.7)	94 (55.3)	
	≥2 shares	142 (53.8)	122 (46.2)	
Vegetable consumption	Rarely	2 (50)	2 (50)	0.205^b^
	<3 shares	83 (44.6)	103 (55.4)	
	3-5 shares	135 (53.8)	116 (2.46)	
Fruit consumption	Rarely	3 (20)	12 (80)	0.005^a^
	2 shares	68 (43.9)	87 (56.1)	
	2-4 shares	149 (55)	122 (45)	

^a^Pearson chi-square; ^b^ Fisher’s exact test; ^c^ Independent *t* test.

 Based on Table 2, the overall TIPI scores were not different between controlled and uncontrolled BP, although the mean individual trait score of extraversion was higher among those with controlled BP, while the mean individual trait scores of agreement, conscience, and emotional stability were higher among those with uncontrolled BP. Also, the mean IPAQ score was higher among those with controlled than uncontrolled BP, *P*<0.001. Also, based on χ^2^ tests, the control of BP was found to vary with the level of PA status, *P*<0.001.

**Table 2:** Distribution of personality traits and physical activity in two groups of patients with controlled and uncontrolled blood pressure

**Variable**		**Blood pressure control status**				***P*** **value** ^a^
		**Yes**		**No**		
		**Mean (SD)**	**Max-Min**	**Mean (SD)**	**Max-Min**	
Extraversion		(1.85) 8.9	2-14	(1.75) 8.32	2-14	0.001
Agreement		(1.52) 8.24	3-12	(1.5) 8.63	2-13	0.007
Conscience		(1.3) 8.02	5-14	(1.47) 8.36	4-14	0.010
Emotional stability		(1.62) 8.15	4-12	(1.88) 8.51	4-14	0.030
being open		(1.62) 8.56	3-14	(1.7) 8.73	3-14	0.283
Total instrument score		(4.94) 41.90	28-53	(5.07) 42.57	28-62	0.155
Physical activity score		3071.01 (39.16)	82-39216.60	1667.59(2880.25)	0-18186	<0.001
		**Number (%)**		**Number (%)**		
Personality characteristics	Week (10-30)	1 (0.50)		1 (0.50)		0.312^b^
	Medium (30-50)	199 (90)		207 (94.1)		
	Strong (50-70)	21 (9.5)		12 (5.5)		
Physical activity status	Inactive	51 (23.2)		116 (52.7)		<0.001
	Low activity	97 (44.1)		69 (31.2)		
	Active	72 (32.7)		35 (15.9)		

SD, standard deviation. ^a^ Independent sample *t* test; ^b^Pearson chi-square.

 Based on Table 3, the univariate analyses showed that the risk factors for uncontrolled BP were age ≥60 years, smoking, Grade-1 and Grade-2 obesity, WC ≥90 cm levels of table salt use, levels of consuming non-liquid plant-based oils, rare consumption of vegetables, levels of fruit consumption, and low. The only protective factor for control of BP was primary education (OR 0.59 [CI: 0.38, 0.92]) (Table 3). Based on multivariate analyses, the risk factors for uncontrolled BP were age ≥60 years, smoking, levels of income, Grade 1 and 2 obesity (, rare fruit consumption, agreement and conscience personality traits. Also, the only protective factors for control of BP were extraversion personality trait (Table 4).

**Table 3:** Univariable (unadjusted) logistic regression model for factors association with uncontrolled hypertension

**Variable**	**Category**	**Unadjusted**	
		**OR (95% CI)**	***P*** value*
Gender	Female	Reference category	
	Male	1.06 (0.70, 1.60)	0.778
Age (y)	<60	Reference category	
	≥60	1.81 (1.24, 2.56)	0.002
Education level	Illiterate	Reference category	
	Primary	0.59 (0.38, 0.92)	0.019
	Secondary	0.58 (0.27, 1.27)	0.344
	University	0.83 (0.50, 1.96)	0.851
Marital status	Single	Reference category	
	Married	1.15 (0.17, 2.84)	0.566
Daily smoking	No	Reference category	
	Yes	2.32 (1.42,3.34)	0.009
Income	< 2 a Month	Reference category	
	2-4 a month	0.85 (0.15, 5.33)	0.478
	>4 a month	1.75 (0.58, 5.35)	0.322
Job	Livestock	Reference category	
	Farmer	2.18 (0.14, 4.36)	0.417
	Manual worker	2.08 (0.12, 5.45)	0.421
	Employee	2.57 (0.18, 6.33)	0.346
	Freelance	1.88 (0.11, 2.78)	0.503
	Housewife	2.00 (0.11, 5.08)	0.427
BMI	Normal	Reference category	
	Overweight	1.65 (1.01, 2.71)	0.045
	Grade 1 obesity	2.53 (1.40, 4.23)	<0.001
	Grade 2 obesity	7.92 (2.79, 12.52)	<0.001
Waist (cm)	<90		
	≥90	2.02 (1.35, 3.02)	<0.001
Use table salt	Seldom	Reference category	
	Sometimes	1.57 (1.05,2.35)	0.011
	Always	3.50 (1.34, 9.14)	0.028
Type of oil consumed	Liquid plant only		
	Oil only solid semi-solid or animal	2.91 (1.45, 3.60)	0.019
	A combination of liquid and solid	2.36 (1.15, 4.85)	<0.001
Consumption of fast food or carbonated beverages	2 a week	1.58 (0.85, 2.91)	0.146
	1-2 a month	1.16 (0.64,2.12)	0.614
	Rarely / Never	Reference category	
Daily milk and dairy consumption unit	≥2 shares	Reference category	
	<2 shares	2.91 (0.55,.27)	0.207
	Rarely	1.44 (0.98,.12)	0.065
Vegetable consumption unit per week	3-5 shares	Reference category	
	<3 shares	0.86 (0.67,1.10)	0.880
	Rarely	1.16 (0.16,8.39)	0.028
Fruit consumption unit per week	2-4 shares	Reference category	
	2 shares	1.56 (1.05, 2.32)	0.016
	Rarely	4.88 (1.35,17.70)	0.028
Extraversion		1.20 (1.04,1.38)	0.001
Agreement		0.84 (0.75, 0.93)	0.008
Conscience		0.85 (0.73, 0.95)	0.011
Emotional stability		0.88 (0.71, 0.96)	0.031
Physical activity status	Active	Reference category	
	Low activity	4.68 (2.78,.78)	<0.001
	inactive	1.46 (0.88,.43)	0.142

Abbreviations: BMI, body mass index; OR, Odd ratio; CI, Confidence interval.

**Table 4:** Multivariable (adjusted) logistic regression model for factors association with uncontrolled hypertension using stepwise approach

**Variable**	**Category**	**Adjusted**	
		**OR (95% CI)**	***P*** ** value**
Age	<60	Reference category	
	≥60	1.98 (0.70, 1.60)	0.014
Physical activity status	Active	Reference category	
	Low activity	1.78 (0.92, 3.41)	0.084
	Inactive	6.11 (3.04,12.27)	<0.001
Marital status	Single	Reference category	
	Married	1.67 (0.89, 3.13)	0.108
Daily smoking	No	Reference category	
	Yes	3.27 (1.56, 6.89)	0.002
Income	< 2 a Month	Reference category	
	2-4 a month	1.70 (0.92, 3.14)	0.088
	>4 a month	6.32(1.57,11.49)	0.009
Type of oil consumed	Liquid plant only	Reference category	
	A combination of liquid and solid	2.01 (1.15, 3.53)	0.014
	Oil only solid semi-solid or anima	2.77 (1.12, 6.84)	0.028
Use table salt	Seldom	Reference category	
	Sometimes	1.52 (0.91, 2.53)	0.112
	Always	2.95 (0.92, 9.46)	0.068
Fruit consumption unit per week	≥2 shares		
	Seldom	5.95 (1.24, 12,1)	0.009
BMI	Underweight and normal	Reference category	
	Grade 1 obesity	2.29 (1.28, 4.09)	0.005
	Grade 2 obesity	7.11 (2.21, 12.52)	0.001
Waist	<90 cm	Reference category	
	≥90 cm	1.99 (1.14, 3.45)	0.015
Agreement		1.26 (1.07,1.48)	0.007
Conscience		1.21 (1.02, 1.44)	0.028
Extraversion		0.85 (0.73, 0.97)	0.020

Abbreviations: BMI, body mass index; OR, Odd ratio; CI, Confidence interval.

## Discussion

 HTN as a disease condition is known since centuries; yet there are systematic struggles that continue to manifest. For instance, the lack of adequate awareness, prevention, control, and mitigation may be noted. Till recently, about half of the world’s adult population is likely to have non-optimal BP levels.^2^ Within this enormous at-risk population pool and from the standpoint of epidemiology and pathophysiology, there are sub-groups with particular characteristics that require special focus, such as older adults. Older adults present unique challenges, such as health, dietary, nutritional, lifestyle, emotive, etc. (e.g., fragility, neuroticism, reduced mobility). These factors may affect their self-efficacy, self-concept, emotional stability, and general ability towards useful health behaviors.^22,23^ Moreover, HTN has a fairly large “web of causation”; thus, any prevention or control mechanisms must be derived thru’ multi-level causal inferences.

 Based on our univariate analyses, the only protective factor for control of BP was primary education. With education comes greater health care awareness that may one help to overcome risks related to HTN, such as low physical activity (PA). Compared to other chronic disease conditions, such as diabetes, HTN and its risk factors are likely to be relatively less known to people with poor education, as these typically develop gradually over many years. Several studies have demonstrated a negative association of cardiovascular disease morbidity and mortality with education.^24^ Since, regions would have poorly educated people, we stress the importance of a sound health policy able to reach out to this group, to make them better aware of HTN, as many such people may go un- or in-adequately treated.

 Based on our multivariable analyses, we found that the risk for uncontrolled BP was related to demographic, lifestyle and personality factors. The problems in HTN control beyond 60 years of age could symbolize a more general gradual temporal increase in the severity of HTN^25^ due to, for instance, increased arterial stiffness, obesity, elevated total cholesterol and low high-density lipoprotein levels, etc. But, in our study, the OR of age factor for BP control was not high, probably because our older adults were near about 60 years of age; and the age effects probably manifest further down the age. Others have also shown that, in general, BP control becomes difficult to achieve with increasing.^23^ Lifestyle modifications are often projected as the only or a cornerstone treatment for controlling HTN in older adults, either with or without active.^13^ In our study as well, lifestyle factors such as rare fruit consumption and Grade-2 obesity were found to yield fairly high ORs; meaning that lifestyle factors are critical in achieving adequate BP control among older adults. However, one also needs to evaluate here about the possibility of challenges in adequate uptake of recommended lifestyle modifications by older adults. Older adults have limitations, such as an age-related decline in mobility, emotional instability, etc., and their day-to-day lifestyle has been set through habitual cues and practices formed over the years. So, it may not be reasonable to anticipate that older adults may swiftly adopt required lifestyle changes. This difficulty could be one of the reasons behind the intricacy in achieving BP control among older adults.^26^ So, we believe that interventions on lifestyle modifications must start early in age and be also integrated with mental health or behavioral interventions.

 Also, part of the problem in assuring uptake of lifestyle modifications for achieving optimal BP control in one’s later ages can be viewed thru’ personality traits as well. For instance, we found that the traits of extraversion were protective for BP control. So, maneuvering one’s awareness of the “impact that their own behavior would have on themselves and those around them” may help to devise suitable personality-based educational actions. Others have also shown that personality traits are modifiable and are moderators of intervention effects.^27^ Evidence is consistent with our findings that revealed extraversion as being associated with BP control.^10^ For instance, individuals who are more extraversion are more likely to do regular physical activity, have regular sleep patterns, have adequate sleep,^28^ tend to be less sedentary,^26^ have more peak aerobic capacity,^29-31^ and tend to engage in behaviors with favorable health and social consequences.^32^ Muslims are instructed by *The Holy Quran* to do “dose-based” daily usual religion practice. Religion is likely to provide sustainable population health benefits thru’ many possible ways, such as promoting abstinence, discipline, positive attitude and knowledge towards health maintenance, etc., as it does not require to uptake difficult lifestyle changes.^33^ Usual religion practice is more pertinent among older adults since older adults are more inclined to seek health and welfare benefits through practice of religion.^34,35^

 Our study has few limitations. For instance, our sample had more females than males However, higher presentation of females in our study may help evade misconceptions that females, especially of the Muslim World, are less likely to seek or access care, at least based on our study. We used case-control design, and calculated odds ratios and effect sizes. Although, odd ratios and effect sizes are standard measures, yet we do not make any cause-effect assertions. Nevertheless, our study provides adequate meat to devise interventions based on factors that we explored in this study.

## Conclusions

 To conclude, several demographic, lifestyle and personality factors were associated with uncontrolled HTN. We recommend to policy-makers to integrate our findings for specific policy-making actions; e.g., early-age personality-based educational interventions through health centers. We also suggest that clinicians make use of personality traits factor as an additional marker of environmental susceptibility during usual chronic care for screening of high-risk subjects and outcome improvement. The role of other more sustainable mechanisms in the control of HTN such as the usual religion practice must be substantiated. We believe our work provides the required knowledge to design comprehensive HTN prevention programs by taking into account the multi-level causality approach.

## Acknowledgments

 We acknowledge the support and participation of health care centers staff and patients.

## Authors’ contributions

 All authors read and approved the final manuscript. AR, NG and AM made contributions to conception and design, acquisition of data, or analysis and interpretation of data. AK, ZJ, FSH and DB analyzed and wrote the manuscript and revised it critically for important intellectual content and edited the manuscript. Finally, all authors agreed to be accountable for all aspects of the work in ensuring that questions related to the accuracy or integrity of any part of the work.

## Funding

 We thank the Research Vice-Chancellor and Tabriz Health Services Management Research Center, Tabriz University of Medical Sciences for the financial support (grant number: 65070).

## Ethical approval

 Ethical approval was obtained from the ethics review committee of Tabriz University (IR.TBZMED.REC.1399.875) with following registering code: 65070. All patients were recruited after written informed consent.

## Competing interests

 The authors declare that there is no conflict of interest.
